# TLR/NF-κB activation in vitro as a tool in the assessment of occupational bioaerosol exposure and health risks in wastewater treatment plants

**DOI:** 10.1038/s41370-026-00880-9

**Published:** 2026-04-20

**Authors:** Anna Jacobsen Lauvås, Anne Straumfors, Sarah Alsaedi, Pål Graff, Mette Myrmel, Anani K. Afanou

**Affiliations:** 1 National Institute of Occupational Health, Oslo, Norway; 2 Virology Unit, Department of Polyclinical Science. Faculty of Veterinary Medicine, Norwegian University of Life Sciences, Ås, Norway

**Keywords:** Air sampling, Virus, Bacteria, WWTP, Innate immune system, Endotoxin

## Abstract

**Background:**

Wastewater treatment plant (WWTP) workers are exposed to biological hazards, including bacteria, fungi, and viruses with pathophysiological properties, posing a risk to workers’ health. Due to the complexity of WWTP bioaerosol composition, exposure assessment is challenging, and new methods are needed for a more holistic evaluation.

**Objectives:**

(1) Characterize bacterial exposure in three Norwegian wastewater treatment plants (WWTPs), (2) Assess the bioaerosol’s and cultivated viruses’ ability to activate toll-like receptors in vitro, and (3) Elucidate adverse health effects in WWTP workers.

**Methods:**

Inhalable bioaerosol fractions were collected with personal and stationary sampling, and endotoxin levels and 16S rRNA gene as a biomarker for bacteria were quantified. The inflammatory potential of the bioaerosol was assessed in vitro with HEK-Blue reporter cells, and we investigated the applicability of these cells for viral ligand detection by exposing them to cultivated Influenza A virus, Adenovirus F/40, *E. coli* phage MS2, and bovine Coronavirus. We used self-reported questionnaire data for an observational health assessment.

**Results:**

TLR2 and TLR4 were activated in 85 and 91% of bioaerosol samples and activation correlated with airborne endotoxin levels. Airborne bacteria were present in all samples, with higher levels in personal (2.7 × 10^4^ gc/m^3^) versus stationary samples (1.3 × 10^4^ gc/m^3^). Endotoxin levels ranged from <LOD to 4800 EU/m^3^ in stationary, and <LOD to 920 EU/m^3^ in personal samples, with median levels of 54 and 47 EU/m^3^, respectively. Workers reported a higher prevalence of respiratory symptoms, skin dryness, diarrhea, and fever than unexposed controls. No bioaerosol sample activated TLR3 and 7, while moderate activation was observed by bovine Coronavirus.

**Significance:**

Workers are continuously exposed to high levels of airborne bacteria levels of endotoxins exceeding 90 Endotoxin units/m^3^, which can induce a TLR2- or TLR4/NF-κB immune response. This exposure occurs throughout their workday, independent of workstations and workers’ tasks.

**Impact:**

- This study highlights the continuous exposure of WWTP workers to bioaerosols with pathophysiological properties, inducing TLR2- and TLR4-NF-κB immune responses. This effect occurs regardless of workstations and workers’ tasks, despite median endotoxin levels remaining below 90 EU/m³. The presence of airborne bacteria and bioaerosols with inflammatory potential may contribute to workers’ health symptoms, and our results highlight the importance of a holistic exposure assessment. We further demonstrate that HEK TLR reporter cell lines provide an innovative method to assess bioaerosol exposure, though they may underestimate exposure to viral-laden bioaerosols.

**Figure:**
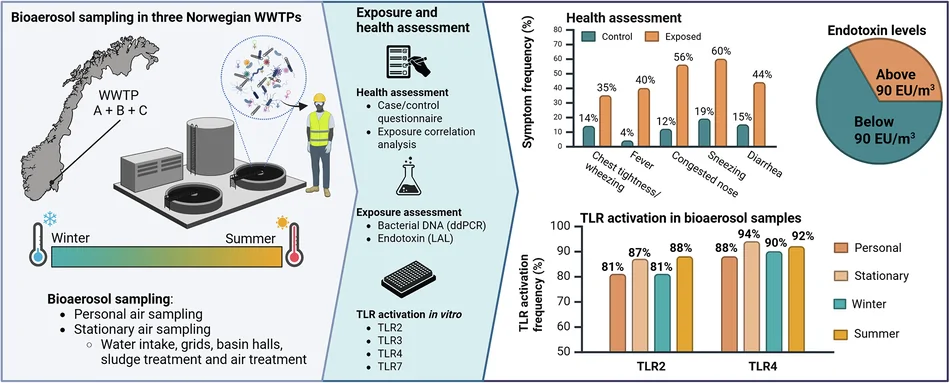

## Introduction

Urban wastewater is a potentially major water pollution source and a carrier of various microbes, including viruses, fungi, and bacteria [1–3]. Wastewater treatment plant (WWTP) workers, therefore, play a crucial role in ensuring public and ecological health. However, bioaerosols generated during wastewater treatment contain microbes, chemical constituents, and organic/inorganic matter, posing an inhalation risk [4–6]. WWTP workers have an elevated risk of gastroenteritis, hepatitis A, respiratory issues (e.g., chronic bronchitis, chest wheezing, and reduced lung function), and sensory dysfunction (smell and taste) [7–9]. Early epidemiological studies suggested higher risks of respiratory, gastrointestinal, and neurological symptoms for workers exposed to dry sludge and untreated wastewater [10]. Treatment processes such as sludge aeration and thickening are linked to increased bacterial levels and species richness [6, 11], indicating bacterial exposure may contribute to these risks. Bacterial exposure in WWTPs varies widely, ranging from 300 to 4.9 × 10^6^ bacterial cells/m^3^ air in Norway [12, 13] and 107 to 8085 colony-forming units (CFU)/m^3^ in Denmark [14], and the overall link between bacterial exposure and work-related symptoms remains inconclusive [12, 13]. Endotoxin, a Gram-negative bacterial cell membrane component, is commonly detected in WWTP bioaerosols [12, 14–18] and high levels are associated with respiratory and flu-like symptoms [9, 19–22], whereas low levels in Danish WWTPs (geometric mean 9.2 EU/m^3^) were considered non-hazardous [14]. A health-based occupational exposure limit (OEL) of 90 endotoxin units (EU)/m^3^ air has been suggested for long-term exposure, based on the no observed effect level for respiratory symptoms [23].

WWTP bioaerosols may also contain both human-pathogenic and non-pathogenic viruses, such as bacteriophages and plant viruses. The bioaerosols, as a complex mixture of bacteria, fungi, and viruses, may contain various pathogen-associated molecular patterns (PAMPs) recognized by human pathogen-recognition receptors such as toll-like receptors (TLRs). The TLRs recognize specific PAMPS, such as endotoxin and viral glycoproteins (membrane TLR4), internalized viral nucleic acids (endosomal TLR 3 and 7), bacterial flagellins (membrane TLR5) and viral/bacterial unmethylated CpG DNA (endosomal TLR9) [24–27]. TLR2 (membrane-bound) recognizes a wide variety of PAMPs, including peptidoglycans (fungi), zymosan (yeast), glycoproteins (viruses), and lipoproteins (bacteria, mycoplasma, viruses) [24, 28–31]. Duerkop and Hooper hypothesized that bacteriophages may become internalized into mammalian cells via phagocytosis or through association with commensal bacteria. Once internalized, their PAMPs could be recognized by TLR3, 7, or 9, cGAMP synthase (DNA), or RIG-1 (RNA) [32]. Activated TLR receptors could lead to the release of NF-κB, and production of Interferon Regulatory Factor 3 (IRF3) and IRF7, followed by the potential production of Interferon β (IFN-β) and pro-inflammatory cytokines [32, 33], leading to an inflammatory response. Exploiting PAMP recognition is a promising approach to study complex bioaerosols with unknown composition, and this approach has been successfully implemented in waste sorting plants [34] and livestock farms [35] settings with highly diverse bioaerosols. To our knowledge, this approach has not been used for WWTP bioaerosols.

This study is part of a larger project where we aim to characterize airborne exposure to viral- and bacterial-laden bioaerosols in Norwegian WWTPs. In the present study, we aim to (1) quantify bacterial load and endotoxins in bioaerosols from three Norwegian WWTPs focusing on seasonal, plant, sampling approaches (personal and stationary) and workstation variations, (2) elucidate the bioaerosol’s ability to induce NF-kB response via TLR 2, 3, 4, and 7 activation, and (3) evaluate associations between bioaerosol exposure and adverse health effects in WWTP workers.

## Materials and methods

### Study sites

Air sampling was performed in three WWTPs (A, B, and C) from December 2022 to June 2023. Plant A and B process wastewater from Oslo city and the surrounding municipalities, with treatment capacities of 3100 and 2700 L/s and 500,000 and 800,000 person equivalents (PE), respectively. The size of a wastewater treatment plant in PE (the organic biodegradable load per day) is determined based on the maximum weekly load discharged to overflow, treatment facilities, or outlet points during the year, excluding unusual conditions such as heavy rainfall [36, 37]. Both plants provide tertiary wastewater treatment (mechanical, chemical, and biological cleansing) and produce biogas from sludge. Oslo’s sewage systems are interconnected, and wastewater can be redirected between plants A and B when exceeding maximum capacity. Plant C, a smaller facility further south in the Oslo fjord, provides secondary wastewater treatment (mechanical and chemical), with a treatment capacity of less than 10,000 PE, and stores sludge prior to disposal. All plants have sealed grid stations and covered treatment processes. Plant B’s grit pit, adjacent to the grid station, is covered, but not hermetically sealed. Plant C’s wastewater intake connects directly to the grid section. See Fig. S1 (supplementary) for representative images of the grid section in plants A, B, and C. Norwegian law mandates protective measures for WWTP workers, including employer-funded vaccinations (tetanus, polio, hepatitis A and B). All exposed workers wear specialized workwear, including safety goggles and protective clothing, with laundry provided at the plant. Workers in Plants A and B use disposable dust masks, while the Plant C worker uses a reusable full mask for respiratory protection. Workers in plants A and B are required to wash their hands and change clothes upon entering clean areas in the administrative building (cafeteria, offices). Both plants had implemented semi-dirty zones in the administrative building and control rooms within the facility, with less stringent requirements. Plant C offers in-house laundry but has not implemented additional hygiene practice rules due to its small size.

### Study population

Potentially exposed workers were identified in cooperation with the health and safety managers at each WWTP, based on their proximity to wastewater or sludge through their work. Given the semi-dirty zones, only a few individuals were considered completely unexposed. Accordingly, unexposed controls were recruited from office personnel at waste sorting plants [38], and one from the management at plant C. Participation was voluntary, and informed, written consent was obtained. A total of 22 controls and 20 exposed workers participated, with some participating on two occasions (four controls and six exposed). At each air sampling occasion, exposed workers provided information on daily work conditions, time spent in the treatment facilities, workstations, specific tasks, duration, use of protective equipment, and handwashing frequency.

### Air sampling campaigns

We obtained permission for sampling twice in winter and summer at Plant A, twice in winter and once in summer at Plant B, and once in winter at Plant C. Samples collected from December 2022 to March 2023 were designated as winter due to consistently cold conditions, and all summer samples were from June 2023. Given the implemented safety measures to reduce worker exposure from hands and work clothes, our sampling focused solely on inhalation exposure. For stationary measurements, we prioritized workstations with direct contact with wastewater or sludge or with potential for aerosolization during manual labor (Table S1). For personal measurements, we equipped workers with samplers within 30 cm of their mouths. Both personal and stationary sampling spanned a full shift of approximately 4–6 h. Temperature and humidity data from selected workstations are available in the supplementary materials of Lauvås et al. [39].

#### Endotoxin sampling and quantification

Endotoxins were sampled on glass fiber filters (25 mm, pore size 1 µm, GF/A, Cytiva, US) mounted in inhalable PAS-6 cassettes [40]. Samplers were connected to GSA (GS5200, GSA Messergerätevau, GmbH, Germany) or Apex pumps (Apex2 standard, Casella solutions, UK), operating at an airflow of 2 L/min (±10%). The dust suspension was extracted as previously described [38]. Filters were extracted in 0.05% Tween-20/endotoxin-free water (5 mL) in pyrogen-free glass tubes by orbital shaking (1000 rpm, 1 h). The extract was centrifuged (1000 × *g*, 15 min), and the supernatant (2 mL) was transferred to a new pyrogen-free glass tube, and centrifugation was repeated. One mL of the supernatant was aliquoted and stored at −20 °C until analysis or immediately assayed in parallel for endotoxin units (EU) with the Limulus Amoebocyte (LAL) kinetic-QCL assay (Lonza Ltd., Basel, CH), following the manufacturer’s instructions. The limit of detection (LOD) was 0.5 EU/filter. Background EU in field blanks was subtracted, and samples below the LOD (*n* = 3, 6% of the total dataset) were replaced with the LOD/√2 for the statistical analysis [41].

#### Bacterial sampling and 16S rRNA gene quantification by ddPCR

Microorganisms were sampled as previously described [39]. Hydrophilic polycarbonate track-etched filters (PCTE, Pore size 1 µm, 37 mm, GVS, Italy) were mounted in autoclaved filter holders in conical inhalable sampler (CIS) cassettes (JS Holdings, Hertfordshire, UK). The cassettes were cleaned with soap and water and disinfected with 75% ethanol before sample mounting. Each sampler was connected to a calibrated GSA 5200 or Apex2 pump that operated at 3.5 L air/min (±10%). In total, 31 personal (29 unique) and 29 stationary samples were collected. Total Field blanks (*n* = 2) were included in summer, opened briefly in the same room as the air samplers were started, and closed without collecting air. After sampling, CIS cassettes were stored in clean zip-lock bags and on ice and processed immediately upon return to the lab. During the first winter sampling at plant B, duplicates from two workers and four stationary samples were eluted with a protocol modified from Alonso and colleagues [42]. Filters were eluted in 3 mL PBS (1X, pH 7.2, Gibco™/Thermo Fischer, US), inverted 10 times, settled, and inverted again, then submerged in an ultrasonic bath for 5 min (Fisherbrand, FB15052, 50/60 Hz). The supernatant was immediately aliquoted and lysed in a PM1 lysis buffer with 2% 2 M dithiothreitol (DTT). Total nucleic acids (tNA) from the remaining samples (*n* = 56) were lysed with 1 mL PM1 lysis buffer applied directly onto the filter. All lysed samples were stored at −20 °C until tNA extraction using the AllPrep Power Viral DNA/RNA Kit (Qiagen, Hilden, Germany) in 50 µL of water, following the manufacturer’s instructions, with a final storage of tNA at −80 °C. The isolation kit was chosen because of its inhibitor removal step, and to enable isolation of RNA and DNA-viruses [39]. Total bacteria measured by the 16S rRNA, hereafter referred to as 16S rRNA, was quantified with ddPCR, using 100 nM forward and reverse primers (sequences in Table S1), 5 µL template tNA diluted 1:100 in nuclease-free water, and 1X EVAgreen supermix (Bio-Rad, US), following the manufacturer’s protocol. Each sample was run at 95 °C for 5 min, followed by 40 cycles at 95 °C for 30 s, 60 °C for 1 min, and 70 °C for 30 s, followed by 5 min at 4 °C. A negative control with no template (NTC) and the threshold was set with a positive control with known presence of 16S rRNA.

#### Total dust sampling

Total dust equivalent to the thoracic bioaerosol fraction was sampled on 25 mm hydrophilic polycarbonate filters (0.8 µm, Merck Millipore, Germany) mounted in antistatic polypropylene filter cassettes (SKC LTD, US) and operated with a GSA or Apex pump at 2 L/min (±10%) [43]. Filters were transferred to clean, nuclease-, and pyrogen-free Falcon tubes using sterile tweezers and stored dry at 4 °C until processing. Dust suspensions were extracted by eluting filters in 5 mL 0.1% PBS/BSA (A8806, Merck Millipore, Germany), sonication for 5 min at room temp followed by orbital shaking (1000 rpm, 1 h). The final dust suspension was aliquoted to pyrogen-free cryotubes and stored at −20 °C until further analysis.

### TLR activation by total dust suspension

We used HEK293 reporter cells (Invivogen, France) expressing hTLR2, hTLR3, hTLR4, or hTLR7 to study TLR/NF-κB pathway activation by PAMPs in bioaerosol samples. These cells release secreted embryonic alkaline phosphatase (SEAP) upon NF-κB activation, which can be measured enzymatically with Quanti Blue solution (Invivogen, France) to assess TLR/NF-κB activation levels. We performed two experiments to assess TLR/NF-κB activation induced by (1) PAMPs in bioaerosol dust, and (2) cultivated human Adenovirus F40 (AdV), Influenza A virus (IAV), bovine Coronavirus (bCoV), and *Escherichia coli* MS2 phage (MS2) (see Table S3 for credits) to evaluate the cell’s ability to recognize viral PAMPs. The proportion of fully or partially degraded viruses in stocks was assessed with capsid-ddPCR.

#### Cell culture maintenance

HEK-Blue™ Null1, hTLR2, hTLR3, hTLR4, and hTLR7 cells (Invivogen, US) were cultured according to the manufacturer’s protocols at 37 °C and 5% CO_2_ in DMEM with GlutaMAX™ (Gibco™/Thermo Fischer, US), 10% heat-inactivated ultra-low endotoxin FBS (BioWest, France), and 100 μg/mL Normocin™ (Invivogen, US). Antibiotics were added for plasmid selection: Zeocin™ (Null1, hTLR3, hTLR7), Blasticidin (hTLR3, hTLR7), and HEK-Blue selection (hTLR2, hTLR4) (all Invivogen). Cells were passaged three times a week at 80–90% confluency by dissociation in PBS (1X, pH 7.2, Gibco™/Thermo Fischer, US). Cell viability was assessed with Via-1 cassettes on a Nucleocounter 200 (Chemometec, Denmark), and experiments were performed at passages 8–15.

#### TLR/NFkB responses induced by total dust suspension and cultivated viruses

Experiments followed previously established methods [44, 45], with some modifications. Cells were seeded at 50,000 cells/well in 96-well plates (Sarstedt, Germany) in cell-line-specific medium. After 24 h, cells were exposed to 20 µL (10% of cell medium) (1) of bioaerosol dust suspension or (2) 1:10 dilution series, starting with the concentrations 8 × 10^6^ gc/µL bCoV, 8 × 10^7^ gc/µL AdV, 4 × 10^6^ gc/µL IAV, or 10^9^ PFU/mL MS2, which are equivalent to multiplicities of infection (MOIs) of 3.2 × 10^3^, 3.2 × 10^4^, 1.6 × 10^3^ and 4 × 10^6^, respectively, assuming 100% infectious virions. Each experiment included negative controls (Endotoxin-free water, Thermo Fischer, US), solvent controls (0.01% BSA-PBS), and untreated controls (cells and medium only), and the cell-line specific positive controls 100 ng/mL LTA (TLR2), LPS (TLR4), Poly(I:C) (TLR3), Imiquimod (TLR7), and TNF-α (Null1 parental control) (all Invivogen). After 24 h, 10% of the cell medium was assayed in a freshly prepared Quanti Blue solution and incubated at 37 °C for 3 h before SEAP activity was measured as absorbance at 649 nm using Synergy Neo2 (BioTEK, Agilent Technologies, USA). Each sample was assayed in technical triplicates and biological quadruplicates (different cell passages). Receptor activation levels were determined by fold change absorbance increase relative to the solvent control. Variation was minimized by removing technical outliers outside the interquartile range (IQR) and excluding remaining measurements with a coefficient of variance >15%. Biological measurements outside the IQR were removed, and the fold change activation of each sample was determined as the average of at least three biological replicates, except 9% of TLR2 (*n* = 5) and 2% of TLR3 (*n* = 1) bioaerosol samples, which were determined from two.

#### Capsid integrity ddPCR with PMAxx

The genome copy numbers of bCoV, IAV, and AdV used to inoculate the HEK reporter cells were determined by regular and capsid integrity ddPCR. After 15 min incubation with 50 µM PMAxx at room temp, this dye binds covalently to DNA/RNA upon activation with blue light, preventing PCR amplification [46]. tNA was extracted with the AllPrep Power Viral DNA/RNA kit as described earlier. Genome concentrations with and without PMAxx treatment was quantified using 5 µL cDNA/DNA template, run with 900 nM forward and reverse primers, 250 nM probe, and 1X Supermix for Probes (no dUTP) (Bio-Rad, US), with initial denaturation at 95 °C for 10 min, followed by 45 cycles of 94 °C for 30 s and 55 °C for 1 min, and a final denaturation at 98 °C for 10 min. Primer sequences are listed in Table S2. Each sample was analyzed without replicates, and each run included a positive and negative control (NTC) for each target.

#### Infectivity assessment of HEK-Blue cells

The permissiveness of HEK-Blue reporter cells to IAV was evaluated using the Null1 parental controls and HEK-Blue hTLR7 cells, which were chosen because they should recognize IAV’s ssRNA. Cells were seeded in 12-well plates at 175 cells/cm² in cell-line-specific medium. After 2 h incubation at 37 °C and 5% CO_2_, the cells were inoculated with 4 × 10^7^ gc/mL IAV at MOI = 63 (assuming 100% infectious virions) or untreated (controls). Cells were harvested at one or 48 h post infection (hpi) and the supernatant at 48 hpi. Before harvesting, the cells were washed three times with PBS. RNA was isolated, and IAV was quantified with ddPCR as described. Cells were monitored and photographed at 10× magnification using a Resolve 4K Hybrid microscope (ECHO, US).

### Workers’ health assessment

A questionnaire was used to survey workers’ respiratory, neurological, and gastrointestinal symptoms. Symptoms were defined as positive if experienced occasionally or frequently. The questionnaire had four parts: (1) background information (sex, age, weight, nicotine intake, underlying diseases, allergies, and prescribed medication), (2) symptoms in the last 12 months, (3) symptoms in the last 7 days, and (4) work-related data (hours spent in the plant, workstations, tasks, and personal protective equipment use) and personal hygiene (handwashing habits), Parts 1 and 2 were completed once per worker; parts 3 and 4 were assessed at each air sampling. Responses from workers participating multiple times were treated as unique responses for summary statistics. Data from unexposed workers, sourced from a previous study, had a slight difference in questionnaires. The questionnaire for exposed workers is provided within the supplementary information; the control questionnaire can be accessed in Eriksen et al. [38] and in supplementary materials.

### Statistical analysis

Data analysis and visualization were performed in R/RStudio (R version 4.3.2) using dplyr/tidyr and ggplot2/cowplot for data management and visualization [47]. Group differences in health responses between exposed and unexposed workers were evaluated with Barnard’s test [48, 49]. No significant dependency was observed with workers’ ID as a random effect in a linear mixed-effect model, and we accordingly estimated odds ratios with a generalized linear model for symptoms due to exposure, controlling for age, BMI, nicotine intake, allergies, asthma, underlying diseases, and handwashing frequency. Models are detailed in Table S4. Differences in aerosolized 16S rRNA and endotoxin concentrations between plants, seasons, and sample types were assessed with Kruskal–Wallis tests and Wilcoxon Signed-rank post-hoc tests. False detection rate (FDR)-adjusted *p* values were considered positive if *p*_fdr_ < 0.05. Positive TLR activation was defined as the average fold change in TLRs exceeding Null1 parental control levels with the Wilcoxon rank sum exact test. Plant, season, and sample type differences in the frequency of TLR-activated samples were determined with Chi [2]. Correlations between bacterial exposure (16S rRNA, endotoxin) and TLR activation and self-reported symptoms were evaluated with Spearman Rank correlation and linear regression on log-transformed exposure data. Infection of IAV was determined with the Wilcoxon Signed-rank test, with *p* < 0.05 considered statistically significant.

### Ethical approval

This study was approved by the Regional Committee for Medical and Health Research Ethics of South-East Norway (REK Case nos. 314217 and 34312). Informed, written consent was obtained prior to participation. All methods were performed in accordance with the relevant guidelines and regulations.

## Results

### Study population—demographics, nicotine habits, and general health status

Exposed and unexposed workers were similar in age, BMI, and nicotine habits (Table 1). The general health status was comparable, except for higher eczema and allergy prevalence among unexposed controls. Exposed workers had more frequent episodes of chest tightness/wheezing in the past year (Table 1). Additionally, 70% of exposed workers experienced short-term flu-like symptoms, and 65% (*n* = 13) reported diarrhea/loose stools, of which five workers had had four or more episodes over the past year. Allergies and eczema on hands or wrists/underarms were less frequent in exposed workers than in unexposed controls (Table 1). The employment duration for exposed workers varied from less than one to 20 years, with an average tenure of 4 years.

**Table 1:** Description of the study population, general health status, and reported symptoms during the last 12 months in exposed workers and unexposed controls.

	Unexposed controls	Exposed
*N* (Females)	22 (*F* = 6)	20 (*F* = 2)
Average age (range) years	42 (29–55)	38 (23–59)
Average height (range) meters	1.78 (1.63–2.03)	1.77 (1.59–1.92)
Average weight (range) kg	84 (56–110)	83 (49–138)
Average BMI (range)	26 (20–31)	26 (19–45)
Daily smokers (past smokers)	3 (8)	5 (6)
Daily snuff users	0	4
Daily E-cigarette users	2	1
General health status		
Underlying disease (medicated/treated)	1 (1)	2 (2)
Asthma (medicated/treated)	0	1 (1)
**Allergy**	**11/22 (50%)**	5/20 (25%)*
Breathing problems	1/22 (5%)	2/20 (10%)
Regular headache	5/22 (23%)	5/20 (25%)
Regular dizziness	1/22 (5%)	1/20 (5%)
Fainted during work	0/22 (0%)	1/20 (5%)
**Eczema hands**	**6/22 (27%)**	1/20 (5%)**
**Eczema wrists/forearms**	**5/22 (23%)**	1/20 (5%)*
Work-related eczema	2/22 (10%)	1/20 (5%)
Symptoms during the last 12 months		
Flu/cold	11/22 (50%)	15/20 (75%)
1–3 times	NA	13/20 (65%)
3–6 times	NA	2/20 (10%)
Short-term flu-like symptoms^a^	0/1 (NA)	14/20 (70%)
Diarrhea/loose stools^a^	1/1 (NA)	13/20 (65%)
1–3 times	NA	8/20 (40%)
4–6 times	NA	3/20 (15%)
> 6 times	NA	2/20 (10%)
Asthma attack	1/22 (5%)	1/20 (5%)
**Chest tightness/wheezing**	**3/22 (14%)**	7/20 (35%)*
Labored breathing without effort	1/22 (5%)	2/20 (10%)
Long-term daily cough with phlegm	1/22 (5%)	2/20 (10%)
Regular morning cough	2/22 (9%)	3/20 (15%)

Health problems are presented as the frequency of reported symptoms (*N*/Total *N*) and percentage (%). Statistically significant differences between groups are indicated with bold text.

*NA* not applicable, *F* female.

**p < 0.01, *p < 0.05, Barnard’s Test.

^a^Response from only one control, plant C.

As few workers participated from each WWTP, the exposed workers were treated as one group. Compared to unexposed controls, exposed workers had higher odds ratios (ORs) for work-related dry skin, fever, congested nose, sneezing, and diarrhea, with ORs of 4.3, 14.7, 54, 5.4, and 6, respectively (Table 2). An increased OR for congested nose was also associated with higher BMI (OR: 1.7, 95% CI: 1.22–2.75), with no significant interaction between exposure and BMI (Table S4). The prevalence of headache and nausea/vomiting was two and three times higher in exposed workers, though not statistically significant.

**Table 2:** Self-reported symptoms during the last 7 days before exposure assessment.

Symptom	Unexposed controls		Exposed	
	*N*/Total (%)	Odds (95% CI)	*N*/Total (%)	OR (95% CI)
Unusual tiredness	7/26 (27%)		8/25 (32%)	
**Dry skin**	**6/26 (23%)**	**0.35 (0.13–0.85)**	15/25 (60%)*	**4.3 (1.3–16)**
Hand eczema	3/26 (12%)		2/25 (8%)	
Headache	5/26 (19%)		10/25 (40%)	
**Fever**	**1/26 (4%)**	**0.05 (0–0.22)**	10/25 (40%)**	**14.7 (2.4–284)**
**Congested nose**^**b**^	**3/26 (12%)**	**0 (0–0)**	14/25 (56%)***	**54 (6–1353)**
Runny/itchy/sore/red eyes	3/26 (12%)		5/25 (20%)	
**Sneezing**	**5/26 (19%)**	**0.3 (0.1–0.7)**	15/25 (60%)**	**5.4 (1.6–21)**
Nausea/vomiting	2/26 (8%)		6/25 (24%)	
Loose stools^a^	–		9/25 (36%)	
**Diarrhea**^**n**^	**4/26 (15%)**	**0.1 (0.01–0.36)**	11/25 (44%)*	**6.0 (1.3–43)**

Controls: 22 individuals, 26 responses Exposed: 20 individuals, 25 responses.

Adjusted for BMI (b), daily nicotine intake (n).

Health problems are presented as the frequency of reported symptoms (*N*/Total *N*) and percentage (%). Odds ratios (ORs) are provided for health problems with statistically significant differences between exposed and unexposed controls (highlighted with bold text).

*p < 0.05, **p < 0.01, ***p < 0.001, Barnard’s test.

^a^Not asked in questionnaire for controls.

^b^Adjusted for BMI.

^n^daily nicotine intake.

### Endotoxin and bacterial exposure

**Fig. 1:**
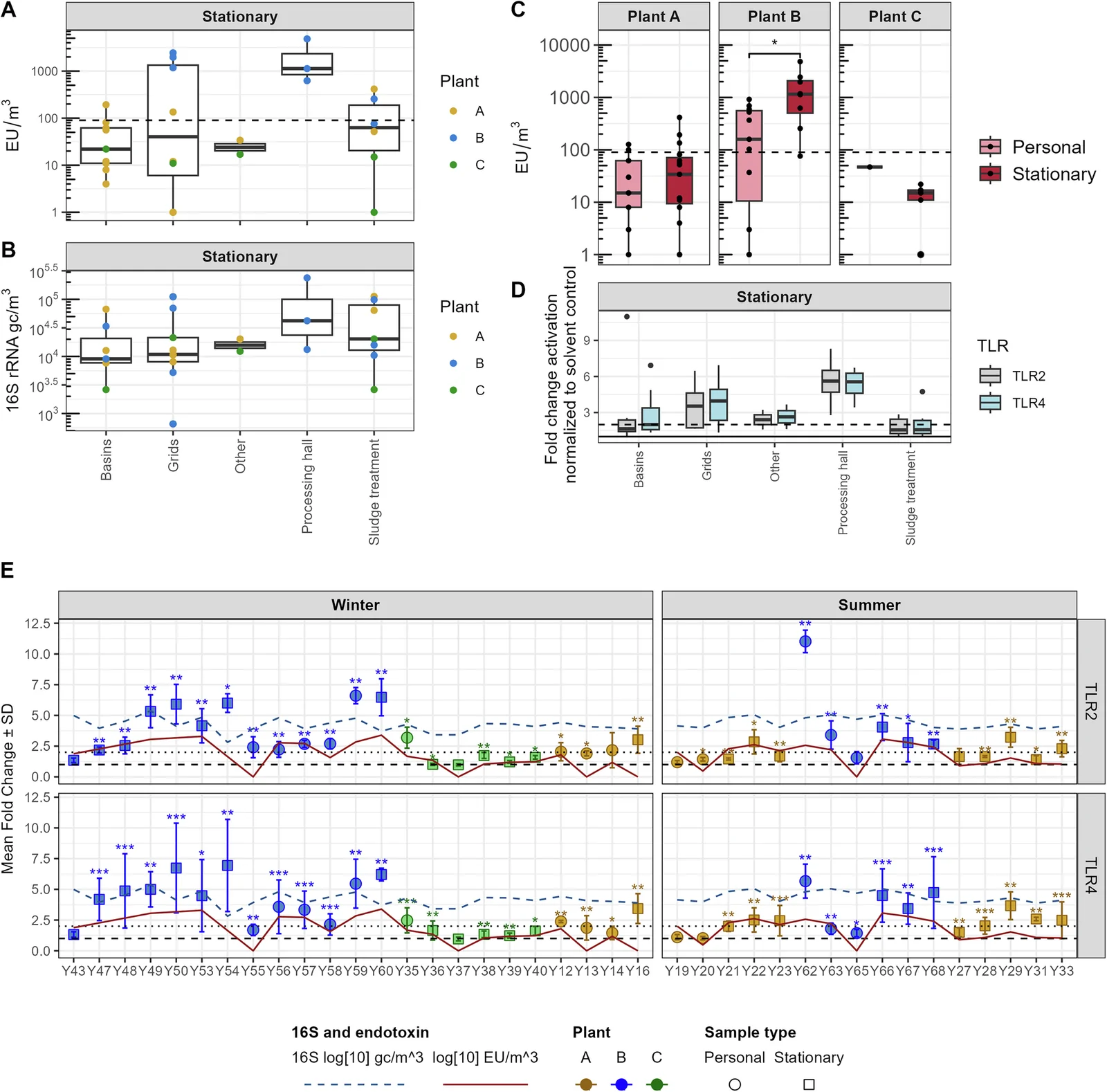
Bacterial (16S rRNA and endotoxin) levels and toll-like receptor activation in bioaerosol samples from three Norwegian WWTPs. **A** Boxplots of airborne endotoxin (EU/m^3^) and **B** boxplots of airborne bacterial DNA (16S rRNA/m^3^) in stationary samples measured at the wastewater basins, grid section, other (air treatment and water intake), processing hall (plant B only), and sludge treatment station, and individual measurements colored by plant. **C** Boxplots of endotoxin measurements in plants A, B, and C, stratified by personal and stationary measurements. The individual measurements are presented as black dots. *p < 0.05. **A**, **C** Dashed lines indicate the health-based recommended limit of 90 EU/m³. **D** Boxplot of TLR2 and TLR4 activation (fold change normalized to the solvent control) in stationary measurements at the wastewater basins, grid section, other (air treatment and water intake), and processing hall (plant B only). Fold change = 2 marked as a dashed line and 1 (no change) as a solid line. **E** TLR2 and TLR4 activation presented as average fold change and standard deviation. Log10-transformed exposure metrics of bacterial DNA and endotoxin are shown as dashed blue and solid red lines. Fold change = 2 marked as a dotted line and 1 (no change) as a dashed line. *p < 0.05, **p < 0.01, ***<0.001 indicate statistically significantly higher TLR activation compared to the Null1 parental control cells.

Endotoxin levels ranged from <LOD to 4800 EU/m^3^ in stationary and <LOD to 920 EU/m^3^ in personal measurements (Table 3). Most stationary measurements (*n* = 18) were below the health-based recommended exposure limit of 90 EU/m^3^. However, among the 10 exceeding this limit, five exceeded 1000 EU/m^3^ and the highest levels were measured at plant B’s processing hall and grid section (Fig. 1A). Considering the overall levels, there was no significant difference between workstations with stationary measurements of endotoxin (*p* = 0.188, Fig. 1A) and 16S rRNA (*p* = 0.361, Fig. 1B). The measured endotoxin levels, however, differed significantly between sampling approaches in plant B when data are stratified by plants. Endotoxin levels by stationary measurements in plant B exceeded both stationary measurements in plant A and C, but also personal measurements collected in plant B (Table 3 and Fig. 1C). To the contrary, there was no significant difference in personal endotoxin levels between plants (Table 3). Still, out of the 9 personal measurements which exceeded 90 EU/m^3^, 7 were observed in plant B and 2 in plant A (Fig. 1C).

**Table 3:** Bacterial exposure in Norwegian wastewater treatment plant measured by 16S rRNA gene copies and endotoxin units per m^3^ air.

Endotoxin (EU/m^3^)		*N*	AM ± SD	GM (GSD)	Observed range (min-max)	Median [Q1–Q75]
Personal	Overall	21	180 ± 270	37 (9.4)	<LOD–920	47 [8–160]
	Plant A	9	40 ± 46	17 (5.0)	<LOD–130	15 [8–62]
	**Plant B**	**11**	**310** ± 330*	**68 (14)**	**1–920**	**160 [20–560]**
	Plant C	1	47	47	–	–
	Summer	6	260 ± 350	50 (16)	1–920	130 [27–320]
	Winter	15	150 ± 240	33 (8.3)	<LOD–690	37 [12–120]
Stationary	Overall	28	490 ± 1100	55 (11)	<LOD–4800	54 [12–300]
	**Plant A**^**b**^	**15**	**72** ± **110**	**23 (6.1)**	**<LOD–420**	**34 [9.5–72]**
	**Plant B**^**a**^	**8**	**1600** ± 1500*	**890 (3.8)**	**76–4800**	**1200 [530–2100]**
	**Plant C**^**b**^	**5**	**13** ± **7.9**	**9 (3.5)**	**1–22**	**15 [11–17]**
	Summer	15	210 ± 320	63 (5.7)	4–1200	62 [12–220]
	Winter	13	810 ± 1500	48 (21)	<LOD–4800	22 [11–1100]
**16S rRNA (gc/m**^**3**^**)**		***N***	**AM** ± **SD**	**GM (GSD)**	**Observed range (min-max)**	**Median [25th–75th]**
Personal	Overall*	**24**	**6.5 × 10**^**4**^ ± **1.4 × 10**^**5**^	**3.1 × 10**^**4**^ **(2.9)**	**8.3 × 10**^**3**^**–7.0 × 10**^**5**^	**2.7 × 10**^**4**^ **[1.2 × 10**^**4**^**–6.1 × 10**^**4**^**]**
	Plant A	9	2.7 × 10^4^ ± 2.7 × 10^4^	2.0 × 10^4^ (2.1)	9.8 × 10^3^–9.4 × 10^4^	1.4 × 10^4^ [1.2 × 10^4^–2.7 × 10^4^]
	Plant B	14	9.3 × 10^4^ ± 1.8 × 10^5^	4.3 × 10^4^ (3.2)	8.3 × 10^3^–7.0 × 10^5^	5.0 × 10^4^ [2.5 × 10^4^–6.4 × 10^4^]
	Plant C	1	1.9 × 10^4^	1.9 × 10^4^	–	–
	Summer	5	5.0 × 10^4^ ± 4.1 × 10^4^	3.4 × 10^4^ (2.8)	9.8 × 10^3^–1.1 × 10^5^	4.9 × 10^4^ [1.4 × 10^4^–6.5 × 10^4^]
	Winter	19	6.9 × 10^4^ ± 1.5 × 10^5^	3.0 × 10^4^ (3.0)	8.3 × 10^3^–7.0 × 10^5^	2.7 × 10^4^ [1.2 × 10^4^–5.5 × 10^4^]
Stationary	Overall*	**29**	**3.6 × 10**^**4**^ ± **5.1 × 10**^**4**^	**1.7 × 10**^**4**^ **(3.6)**	**6.6 × 10**^**2**^**–2.4 × 10**^**5**^	**1.3 × 10**^**4**^ **[9.1 × 10**^**3**^**–4.2 × 10**^**4**^**]**
	Plant A	11	3.0 × 10^4^ ± 3.5 × 10^4^	1.9 × 10^4^ (2.7)	7.7 × 10^3^–1.1 × 10^5^	1.3 × 10^4^ [9.3 × 10^3^–4.2 × 10^4^]
	Plant B	13	5.1 × 10^4^ ± 6.7 × 10^4^	2.1 × 10^4^ (4.8)	6.6 × 10^2^–2.4 × 10^5^	1.6 × 10^4^ [9.4 × 10^3^–7.0 × 10^4^]
	Plant C	5	1.2 × 10^4^ ± 9.1 × 10^3^	8.2 × 10^3^ (2.9)	2.6 × 10^3^–2.1 × 10^4^	1.2 × 10^4^ [2.6 × 10^3^–2.0 × 10^4^]
	Summer	11	3.8 × 10^4^ ± 4.1 × 10^4^	2.2 × 10^4^ (2.9)	7.7 × 10^3^–1.1 × 10^5^	1.3 × 10^4^ [1.1 × 10^4^–5.5 × 10^4^]
	Winter	18	3.5 × 10^4^ ± 5.7 × 10^4^	1.5 × 10^4^ (4.2)	6.6 × 10^2^–2.4 × 10^5^	1.3 × 10^4^ [8.3 × 10^3^–3.1 × 10^4^]

The data are presented as the arithmetic (AM) with standard deviations (SD), geometric mean (GM) with geometric standard deviations (GSD), observed range, and median with 25th–75th percentile.

*p < 0.05, significant difference between personal and stationary measurements.

*Significant difference between personal and stationary concentrations (*p* < 0.05). Statistically significant differences in endotoxin levels among plants are indicated by different lowercase letters; plants sharing the same letter do not differ significantly from each other.

^a,b^Contrasts with significant difference (*p*_fdr_ < 0.05).

Aerosolized 16S rRNA levels were higher in personal versus stationary samples. We observed a trend towards higher levels in plant B’s stationary samples compared to plant A and C (*p*= 0.054), consistent with increased endotoxin levels (Table 3). There was no seasonal difference in endotoxin or 16S rRNA concentrations.

### TLR/NF-kB-induced responses by WWTP bioaerosols

In total, 85 and 91% of bioaerosol samples activated TLR2 or TLR4, respectively. Activation frequencies for TLR2 and TLR4 were similar in personal (81 and 88%, respectively) and stationary samples (87 and 94%, respectively). All personal samples from plant B were positive for TLR4 activation, and 89% for TLR2 activation, versus 67% for both receptors in plant A (Table 4). There was no statistically significant difference in TLR2 and TLR4 activation frequency between the personal and stationary samples, plants, or seasons (Fig. 1E and Table 4). However, personal samples trended towards a higher activation frequency of TLR4 in summer compared to winter (*p* = 0.054). No activation of endosomal TLR3 and TLR7 was observed (Table 4 and Fig. S2).

**Table 4:** Toll-like receptor activation in wastewater treatment plant bioaerosols.

		TLR2	TLR3	TLR4	TLR7
		Positive/*N* (%)	Positive/*N* (%)	Positive/*N* (%)	Positive/*N* (%)
All samples	Overall	40/47 (85%)	0/47 (0%)	43/47 (91%)	0/47 (0%)
	Plant A	15/19 (79%)	0/19 (0%)	17/19 (89%)	0/19 (0%)
	Plant B	20/22 (91%)	0/22 (0%)	21/22 (95%)	0/22 (0%)
	Plant C	5/6 (83%)	0/6 (0%)	5/6 (83%)	0/6 (0%)
	Winter	23/26 (88%)	0/26 (0%)	24/26 (92%)	0/26 (0%)
	Summer	17/21 (81%)	0/21 (0%)	19/21 (90%)	0/21 (0%)
Personal	Overall	13/16 (81%)	0/16 (0%)	14/16 (88%)	0/16 (0%)
	Plant A	4/6 (67%)	0/6 (0%)	4/6 (67%)	0/6 (0%)
	Plant B	8/9 (89%)	0/9 (0%)	9/9 (100%)	0/9 (0%)
	Plant C	1/1 (100%)	0/1 (0%)	1/1 (100%)	0/1 (0%)
	Summer	9/10 (90%)	0/10 (0%)	10/10 (100%)	0/10 (0%)
	Winter	4/6 (67%)	0/6 (0%)	4/6 (67%)	0/6 (0%)
Stationary	Overall	27/31 (87%)	0/31 (0%)	29/31 (94%)	0/31 (0%)
	Plant A	11/13 (85%)	0/13 (0%)	13/13 (100%)	0/13 (0%)
	Plant B	12/13 (92%)	0/13 (0%)	12/13 (92%)	0/13 (0%)
	Plant C	4/5 (80%)	0/5 (0%)	4/5 (80%)	0/5 (0%)
	Summer	14/16 (88%)	0/16 (0%)	14/16 (88%)	0/16 (0%)
	Winter	13/15 (87%)	0/15 (0%)	15/15 (100%)	0/15 (0%)

The data are presented as the number and percentage of bioaerosol samples with statistically significant activation (*p* < 0.05) of TLRs 2, 3, 4, and 7 compared to the Null1 parental control cell line out of the total number of samples, *N*, in plants A, B, and C.

The highest fold change of TLR2/- and TLR4/NF-κB-induced responses were observed in stationary samples collected at the grid station, processing hall (plant B) or air treatment room/water intake (grouped “other” in Fig. 1D) and in personal and stationary measurements from plant B (Fig. 1E). These workstations and plant B were also associated with the highest endotoxin levels, except for the air treatment room/water intake (Fig. 1A). The fold change increase of TLR2 and TLR4 activation significantly and positively correlated with endotoxin levels (*ρ* = 0.62, *p* < 0.001 and *ρ* = 0.66, *p* < 0.001, respectively) (Table S6). Fold change TLR activation increased in a log-linear manner with increasing endotoxin levels; TLR4 by 1.2 ± 0.2 (*p* < 0.001) and TLR2 by 1.4 ± 0.4 (*p* < 0.001). No significant correlation was observed for the levels of airborne 16S rRNA gene copies and TLR activation, although this exposure metric positively correlated with the endotoxin levels (Table S6). Additionally, TLR2 fold change activation strongly and positively correlated with TLR4 (*ρ* = 0.91, Table S6). Previously measured airborne concentrations of Pepper Mild Mottle Virus, Adenovirus, Norovirus GI and GII, and Influenza A virus [39] did not correlate with fold change activation of TLRs 2, 3, 4, or 7 (Data not shown).

#### TLR/NF-kB-induced responses by cultivated viruses

**Fig. 2:**
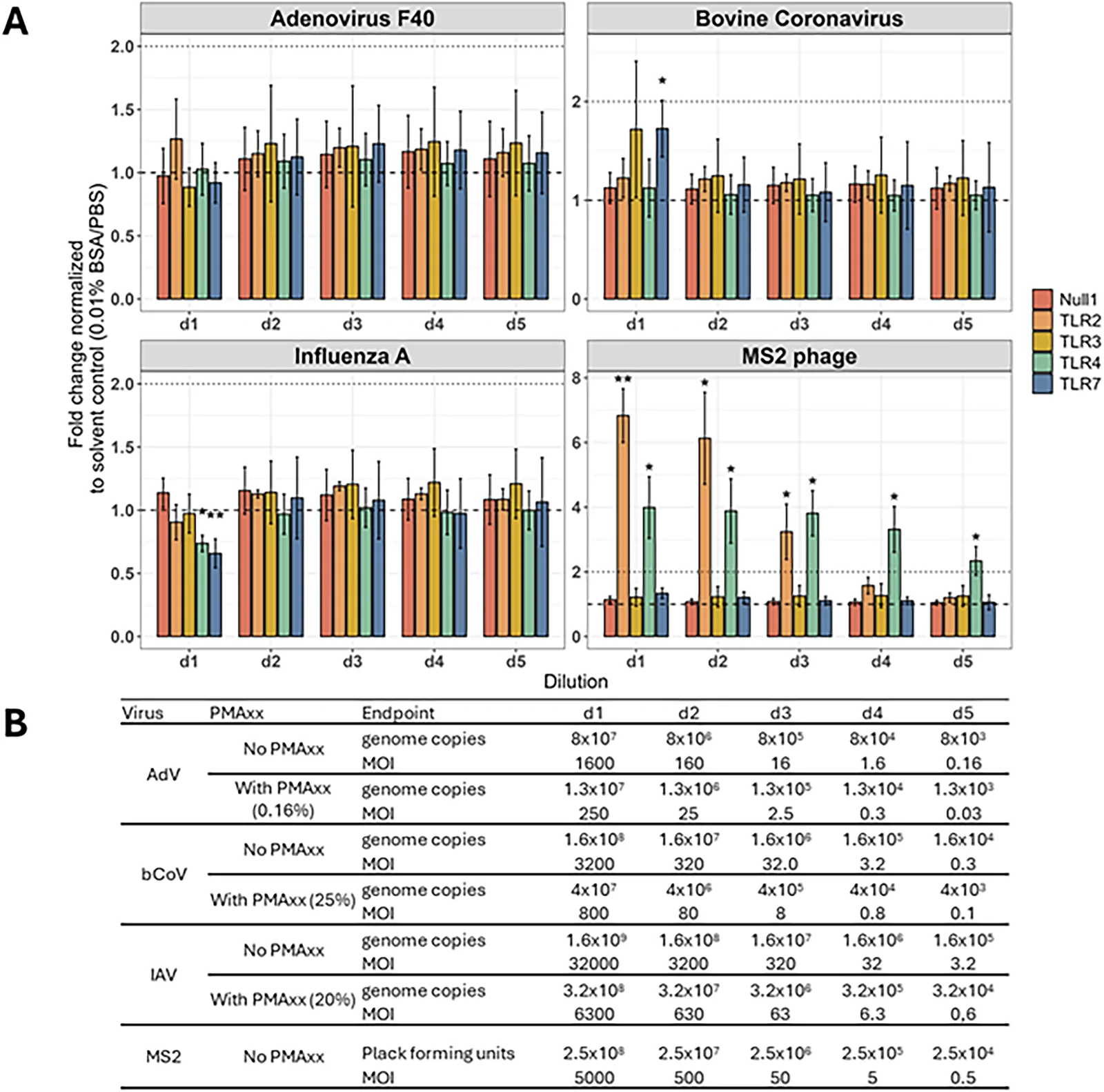
Toll-like receptor activation in TLR 2, TLR3, TLR4, and TLR7 after 24-h incubation with a dilution series of AdV F40, IAV PR8, MS2, and bCoV. **A** TLR activation presented as fold change activation compared to the solvent control (0.01% BSA/PBS) across a dilution series of dilutions d1–d5. *p < 0.05, **p < 0.01, and ***p < 0.001 indicate statistically significant differences for the equivalent sample in the Null1 parental control cells. The *y*-axis is specific for each virus. **B** The concentration of AdV, bCoV, and IAV, determined by ddPCR (no PMAxx) and capsid-integrity ddPCR (with PMAxx), and the concentration of MS2 phage, determined as plaque-forming units, corresponding to each dilution (d1–d5) used in (**A**). MOI multiplicity of infection.

Analysis using capsid integrity ddPCR revealed that 25% (bCoV), 20% (IAV), and 0.16% (AdV) of the genomic copies were associated with intact capsids. TLR2 and 4 were activated in a dose-response manner by MS2, whereas no activation from IAV, AdV or bCoV was observed (Fig. 2A). TLR7 was activated with the highest bCoV concentration of 1.6 × 10^8^ genome copies (d1), which was accompanied by a trend towards an increased TLR3 activation (Fig. 2A). At the highest concentration of IAV, a statistically significant reduced activation compared to the solvent control was observed in TLR4 and TLR7 (Fig. 2A). After 24 h, we observed signs of cytopathic effect such as cell rounding and detachment in Null1, TLR7 and TLR4 cells exposed to IAV (data not shown). We therefore evaluated the infection of IAV in the TLR7 and the parental control Null1 cells.

#### Infection by the Influenza A virus

At 48 hours post infection (hpi), we observed clear morphological changes in TLR7 and Null1 cells inoculated with IAV, including cell rounding and detachment (Fig. S4A). We also observed increased cell-associated IAV from 2.13 × 10^5^ and 6.55 × 10^4^ genome copies at 1 hpi to 3.26 × 10^6^ and 9.72 × 10^5^ genome copies at 48 hpi in Null1 and TLR7 cells, respectively, yielding approximately 15-fold increase in both cell lines (Fig. S4B).

#### Relationship between exposure measurements and workers’ health symptoms

There was no significant relationship between the measured exposure components (endotoxin units,16S rRNA gene copies, TLR2 and TLR4 activation) and the health effects reported by the workers (*p* > 0.05, Table S5).

## Discussion

In this study, we characterized occupational exposure to bacteria with the 16S rRNA gene and endotoxin levels in aerosols in Norwegian WWTPs. NF-κB induced response by the bioaerosol through activation of TLRs 2, 3, 4, and 7 was also assessed and related to the measured bacterial exposure and workers’ self-reported health effects. Finally, the suitability of HEK-Blue TLR2, TLR3, TLR4, and TLR7 expressed in reporter cell lines was assessed for the identification of occupational exposure to viral ligands.

### TLR2 and TLR4 activation potential of the WWTP bioaerosol

To the best of our knowledge, this is the first study to characterize the TLR/NF-κB activation potential of the total dust fraction from WWTP bioaerosol in vitro. The 85 and 91% activation frequencies of TLR2 and TLR4 (Table 4) are approximately three times higher than our previous reports from Norwegian waste sorting plants, where activation was observed in one-third of all samples [34]. The WWTP bioaerosol is a heterogeneous mixture of microbes, particulate matter, and chemical vapors [4], and our results indicate the presence of PAMPs with potential pathophysiological properties. Activation of TLR2 (81%) and TLR4 (88%) by personal samples indicates that workers are exposed to PAMPs through their work, and the high activation frequency in stationary samples indicates a potential for consistent exposure across all plants, independent of workstations and tasks. Due to the WWTP bioaerosol’s complexity and TLRs wide range of PAMP recognition, identification of a specific ligand is not achievable. However, endotoxin levels moderately and positively correlated with fold change activation of TLR2 (*ρ* = 0.62) and TLR4 (*ρ* = 0.66). Compared to our previous studies in waste sorting plants, endotoxin correlated more strongly with TLR2 and TLR4 than those reported by Afanou et al. [44] (*r* = 0.42 and *r* = 0.52, respectively) and were comparable or weaker than those reported by Eriksen et al. (*r* = 0.6 and *r* = 0.8, respectively) [34, 44]. We employed similar sampling and analysis protocols and therefore attribute the observed differences to variations in the bioaerosol composition. The acylation level of endotoxins is critical for the activation of TLR4, and endotoxins with highly acylated lipid A increase the inflammatory response induced by TLR4, whereas low acylated endotoxins are poor TLR4 stimulators [50, 51]. The WWTP bioaerosol microbial composition is diverse, but bacterial species and genera with highly acylated endotoxins, such as *E. coli*, *Pseudomonas*, and *Acinetobacter*, are commonly detected [52].

### Endotoxin and bacteria

Endotoxin levels in personal samples (GM 37 EU/m^3^) exceeded findings from Danish WWTPs (GM 9.2 EU/m^3^) [14] and were seven-fold higher in plant B (median 160 EU/m^3^) compared to previous results from the same plants (median 24 EU/m^3^) [18]. Conversely, levels in plant A were threefold lower (15 EU/m^3^ versus 50 EU/m^3^) [18], possibly due to a recent upgrade of the ventilation system. High 16S rRNA levels indicate substantial bacterial load in both personal (6.5 × 10^4^ gc/m^3^) and stationary (3.6 × 10^4^ gc/m^3^) samples, consistent with previous studies from Chinese, Danish, and Dutch WWTPs [6, 11, 14, 53, 54]. Similar patterns have been observed in other working environments with high bioaerosol generation, such as British composting [55], Norwegian waste sorting [38, 43], and Italian livestock farms [56]. The highest levels of 16S rRNA (2.4 × 10^5^ gc/m^3^) and endotoxin (4800 EU/m^3^) were found close to the wastewater surface in plant B’s processing hall. This aligns with findings from Chinese WWTPs, where the highest bacterial count was found close to the wastewater surface [53]. The higher bacterial DNA load in personal samples compared to stationary ones overall aligns with reports from Danish WWTPs [14] and may result from working in proximity to wastewater or sludge [53], as well as tasks like flushing or the agitation of bacteria on workers’ clothing [57]. The moderate correlation between bacterial load and endotoxin (Spearman *ρ* = 0.48) can be explained by the presence of various bacterial species, including Gram-positive bacteria, which lack endotoxin [6, 52, 58].

The significantly higher endotoxin levels in plant B compared to plants A and C overall could be explained by a higher bioaerosol emission at plant B’s grids station, where we observed 18-fold higher endotoxin levels in plant B (1200–2500 EU/m^3^) compared to plant A (<LOD to 135 EU/m^3^). This could also be linked to differences in the wastewater composition, which has been demonstrated to significantly impact microbial community composition in WWTPs [59]. However, differences in wastewater composition could not explain the 9-fold higher bacterial DNA load in plant B (1.1 × 10^5^ gc/m^3^) compared to plant A (1.3 × 10^4^ gc/m^3^) (Fig. 1A). Considering the fact that both plants treat sewage from the same city, and experienced similar environmental conditions throughout our sampling campaigns [39], other factors may be at play, such as structural and cultural differences between plants. Plant B’s grid station is located immediately next to a grit chamber, which is normally covered with metal plates (see Fig. S1). During our air sampling, these plates were removed due to manual grit removal (sand pumping), resulting in dual exposure from both the grid and grit chamber in Plant B. Additionally, we observed more wastewater and sludge residues on floors and equipment in plant B compared to plants A and C. This may have contributed to increased bacterial growth on surfaces and higher bioaerosol levels due to dust resuspension [60]. Improving routine cleaning practices and implementing automation of treatment processes could reduce worker’s exposure. These findings highlight the importance of considering WWTP layout and workstation design when planning new facilities.

### Health effects

Our data revealed increased risk of dry skin, fever, upper respiratory symptoms (sneezing, congested nose), and diarrhea in WWTP workers, consistent with previous findings from Dutch, Swedish and Norwegian WWTPs [9, 10, 13] and lower respiratory symptoms (chest tightness/wheezing), previously associated with >5–10 years of employment [20]. We did not find a relationship between self-reported health effects and airborne endotoxin levels, likely due to high variability in individual endotoxin measurements and the relatively low levels detected (median 47 EU/m³). Similar findings have been reported in Swiss WWTPs, where endotoxin levels were also not correlated with respiratory symptoms [9, 61]. However, the highest personal exposure in the present study was 920 EU/m³, ten times the recommended limit of 90 EU/m³, and all but three stationary samples contained quantifiable endotoxin levels, indicating continuous exposure. Acute endotoxin inhalation in humans is associated with increased pulmonary secretion, chest wheezing, and cough [62], and a biphasic inflammatory response with neutrophil influx during the first 24 h, followed by macrophage, monocyte, and lymphocyte influx 24–48 h post exposure [62, 63]. Persistent exposure to endotoxin increases airway resistance in mice [64], and an inverse relationship between airborne endotoxin levels and forced expiratory volume (FEV1) in WWTP workers was previously found [18]. The TLR2 and TLR4 activation induced by most air samples indicates that workers are exposed to potentially inflammatory agents. It is worth highlighting that the TLR2- and TLR4-activation occurred in bioaerosol fractions equivalent to the thoracic fraction, which can reach the tracheobronchial and respirable region. Chronic exposure to inflammatory bioaerosols may therefore explain the 35% of workers who reported chest tightness or wheezing in the past year, despite the low measured endotoxin concentrations. Short-term flu-like symptoms (reported by 70% of exposed workers) are consistent with endotoxin-related hypersensitivity reactions [20]. However, as noted by Thorn et al. [10], such symptoms may also result from infections caused by pathogenic bacteria [10]. The 16S rRNA gene is a universal marker that does not differentiate between pathogenic, opportunistic, and commensal bacteria. Several pathogenic and opportunistic bacteria have indeed been detected in WWTP bioaerosols, including *E. coli*, *Klebsiella pneumonia*, *Shigella sp*., *Enterobacter sp*., and others [6, 11, 14, 52, 53], which can cause diarrhea, respiratory tract infections, or fever. Pathogenic viruses have also been detected in WWTP bioaerosols [65, 66], and we previously detected both human adenovirus and norovirus (GGI and II viruses) in personal and stationary samples during the same sampling campaign described in the present study [39]. An in-depth analysis of the microbial composition is beyond our scope, but we cannot rule out pathogenic microbes as potential contributors to the observed symptoms. Interestingly, approximately four times as many unexposed individuals reported eczema on the hands or wrists, and twice as many reported having allergies, compared to those exposed to WWTP bioaerosol. Skin conditions have been proposed as a strong health-based reason for changing jobs; thus, the observed difference may reflect a so-called healthy worker effect [67]. However, despite the lower prevalence of eczema, exposed workers reported significantly more issues with dry skin compared to unexposed controls, which may be due to chemical exposure [4, 68]. Although the causative agents of these symptoms remain unknown, it is worth noting that exposure and symptoms occurred despite implementation of safety measures, such as vaccines, workwear, protective gloves, shoes, glasses, and masks, and the plants’ exposure reduction strategies.

### Viral PAMP recognition in HEK-Blue TLR/NF-κB reporter cells

We previously detected Pepper mild mottle virus, Adenovirus, Norovirus, and Influenza A virus in the WWTP bioaerosols studied in the present study [39] and therefore tested whether HEK-Blue TLR3 and TLR7 cells could recognize viral nucleic acids. Despite the presence of viruses in the bioaerosol samples, no activation was found. This is consistent with a Dutch study on farm bioaerosols, where no activation was observed using the same reporter cells [35]. Furthermore, our dose-response evaluation of cultivated viruses showed no activation, neither by human AdV F40, IAV, nor bacteriophage MS2. Only bCoV induced TLR7 activation at a high concentration (1.6 × 10^8^ genome copies). No such response was observed in the Null1 parental controls, suggesting TLR7/NF-κB activation. However, the required concentration of 1.6 × 10^8^ genome copies is equivalent to a multiplicity of infection of 800 (assuming 25% infectious viruses, determined by capsid-ddPCR) or 3200 (assuming 100% infectious viruses). Such high values may indicate an unspecific reaction, although bCoV variant HECoV-4408 has been confirmed to cross the intraspecies barrier between humans and calves [69, 70]. Our results indicate that the HEK-Blue reporter cells are permissive to bCoV and may recognize internalized viral nucleic acids from mammalian viruses with some resemblance to human-pathogens. However, an MOI of 800 likely exceeds real-life scenarios and has limited practical relevance to occupational exposure in WWTPs.

Of the cultivated viruses in the present paper, only MS2 induced activation of TLR2 (four-fold) and TLR4 (seven-fold). However, the activation may result from remnants from the host, *E. coli*, such as lipopolysaccharide (LPS), and we cannot conclude that the activation results from the bacteriophage itself [71]. Our infectivity assessment confirmed that IAV replicates in the HEK TLR7 and Null1 parental control cells, demonstrated by a loss of and detachment of cells (Fig. S4A) and approximately 15-fold increase in cell-associated IAV in both cell models at 48 hpi (Fig. S4B). This likely accounts for the reduced SEAP levels observed in HEK TLR7 and TLR4 cells inoculated with IAV in Fig. 2A, as the infected cells were undergoing cell death. Nonetheless, we observed no TLR/NF-κB activation in response to IAV inoculation at sub-infectious doses in our dose-response assessment (Fig. 2A). This may be explained by divergent IAV-induced inflammatory responses in the non-respiratory HEK-Blue cells compared to respiratory cells. For example, Bernasconi and colleagues showed that although IAV replicates in several non-respiratory cell lines, including HEK293, NF-κB activation was observed only in pulmonary A549 cells [72]. Through heterodimerization with TLR1 and TLR6, TLR2 should be able to recognize a variety of viral surface proteins [29]. In the present study, we observed no activation of either TLR2 or TLR4, nor TLR3 and TLR7 by the cultivated, mammalian viruses AdV, IAV, and bCoV at sub-infectious doses, which may be explained by viral immune evasion strategies [73, 74]. The E1B protein in AdV suppresses NF-κB activity [75], and the NS1 protein in IAV interferes with NF-κB binding to promoter DNA [76], which will inhibit SEAP production in the reporter cells. As reviewed by Lester and colleagues, viral countermeasures to TLR signaling are common in a wide range of human pathogenic viruses [73] and may result in no TLR/NF-κB response during active replication. It is important to underline, however, that this may not be the case for all human pathogenic viruses. For example, tick-borne encephalitis virus (TBEV) has been shown to induce a TLR/NF-κB response via TLR3, TLR7, and TLR8 activation in HEK-Blue cells in vitro, despite no activation of TLR2 and TLR4 [77]. As reviewed by Colleselli et al. [78], a variety of other co-receptors than TLR1 and 6 are suggested to play an important role in TLR2 signaling. According to the Human Protein Atlas, HEK293 cells express very low levels of TLR1 and TLR6 [79]. A lack of TLR2 activation of both AdV, IAV, and bCoV may thus result from imbalanced TLR1 or TLR6 expression or other required co-receptors in the HEK-Blue cells. For example, Groot et al. [80] showed that TLR1 was required for TLR2 activation by *Mycoplasma pneumoniae*, but not *Staphylococcus pneumoniae*, whereas TLR2 signaling caused by both bacteria was independent of TLR6. Francisco et al. [81] found that *E. coli* LPS signals exclusively through TLR4, whereas a heterodimer complex of TLR2/TLR4/MD-2 was required for *Ochtrobactrum intermedium* signaling. Further research is warranted to identify which TLR receptors and co-receptors are required for specific PAMP recognition to more fully interpret the results acquired from the HEK-Blue TLR/NF-κB reporter cell model. As a tool in viral exposure assessment, our results indicate that future studies should carefully evaluate the suitability of the model to each human pathogenic virus of interest.

### Limitations

Our epidemiological health assessment may be subject to differential and non-differential information bias due to reliance on questionnaire data for symptoms. The overall study population was quite small, resulting in low power for symptoms that have a weak association with the exposure. Despite these limitations, the reported symptoms are significant and in alignment with the previous literature [4, 7, 10, 19, 20, 82]. The wide 95% CIs of all ORs are likely connected to the small study population, indicating uncertainty regarding the exact OR in exposed workers compared to unexposed controls, and may mask and/or exaggerate effect estimates. The total dust samples intended for cell assay were stored dry on filters at 4 °C until elution, which may have enabled degradation of viral particles and other PAMPs. Additionally, our assessment of individual virus levels shows that virus-initiated TLR/NF-κB activation of the investigated viruses was not feasible in the reporter cells, and it is unlikely that degradation is the main cause for negative viral TLR activation in the bioaerosol suspension.

## Conclusions

This study demonstrated that 85 and 91% of the thoracic total dust fraction of Norwegian WWTP bioaerosols can induce a TLR2- or TLR4/NF-κB inflammatory response, respectively, in vitro. This suggests that WWTP workers risk exposure to bioaerosols with inflammatory potential, regardless of their proximity to high-risk workstations and tasks. Airborne 16S rRNA gene levels were consistently high and higher in personal than in stationary measurements, suggesting that stationary measurements might underestimate workers’ exposure to airborne bacteria. Endotoxin levels varied widely from <LOD to 4800 EU/m^3^, with most measurements below <90 EU/m^3^ (median 47 EU/m^3^) and significant spatial variation between plants. Our results demonstrate that Norwegian WWTP workers are at increased risk of developing both upper and lower respiratory symptoms and that these symptoms may partly be associated with the inflammatory response induced by bacterial exposure throughout their work. Our findings are consistent with previous studies and reveal that respiratory symptoms are common despite exposure mitigation measures such as encapsulation, ventilation, and specialized workwear. Our results indicate that the HEK-Blue TLR/NF-κB reporter cells may be incompatible with PAMP recognition of prevalent human pathogenic viruses, such as AdV F40 and IAV. This highlights the need for new tools to assess occupational exposure to viral-laden bioaerosols effectively.

## Supplementary information

## Data Availability

The exposure and in vitro data underlying this article are available on reasonable request. The data underlying the health assessment is not available due to sensitive personal information.
