# Supplementary material for: TLR/NF-κB activation in vitro as a tool in the assessment of occupational bioaerosol exposure and health risks in wastewater treatment plants
Source: J Expo Sci Environ Epidemiol. 2026 Apr 20;36(5):939–50. doi: 10.1038/s41370-026-00880-9 (PMC13538029; doi:10.1038/s41370-026-00880-9)
Supplement: Supplementary file 1 [file 41370_2026_880_MOESM1_ESM.pdf]

TLR/NF-κB activation *in vitro* as a tool in the assessment of occupational bioaerosol exposure and health risks in wastewater treatment plants – Supplementary information

Anna Jacobsen Lauvås[1,2], Anne Straumfors[1], Sarah Alsaedi[1], Pål Graff[1], Mette Myrmel[2], Anani K. Afanou[1*]

[1]National Institute of Occupational Health, Oslo, Norway

[2]Norwegian University of Life Sciences, Ås, Norway

*Corresponding author: anani.afanou@stami.no

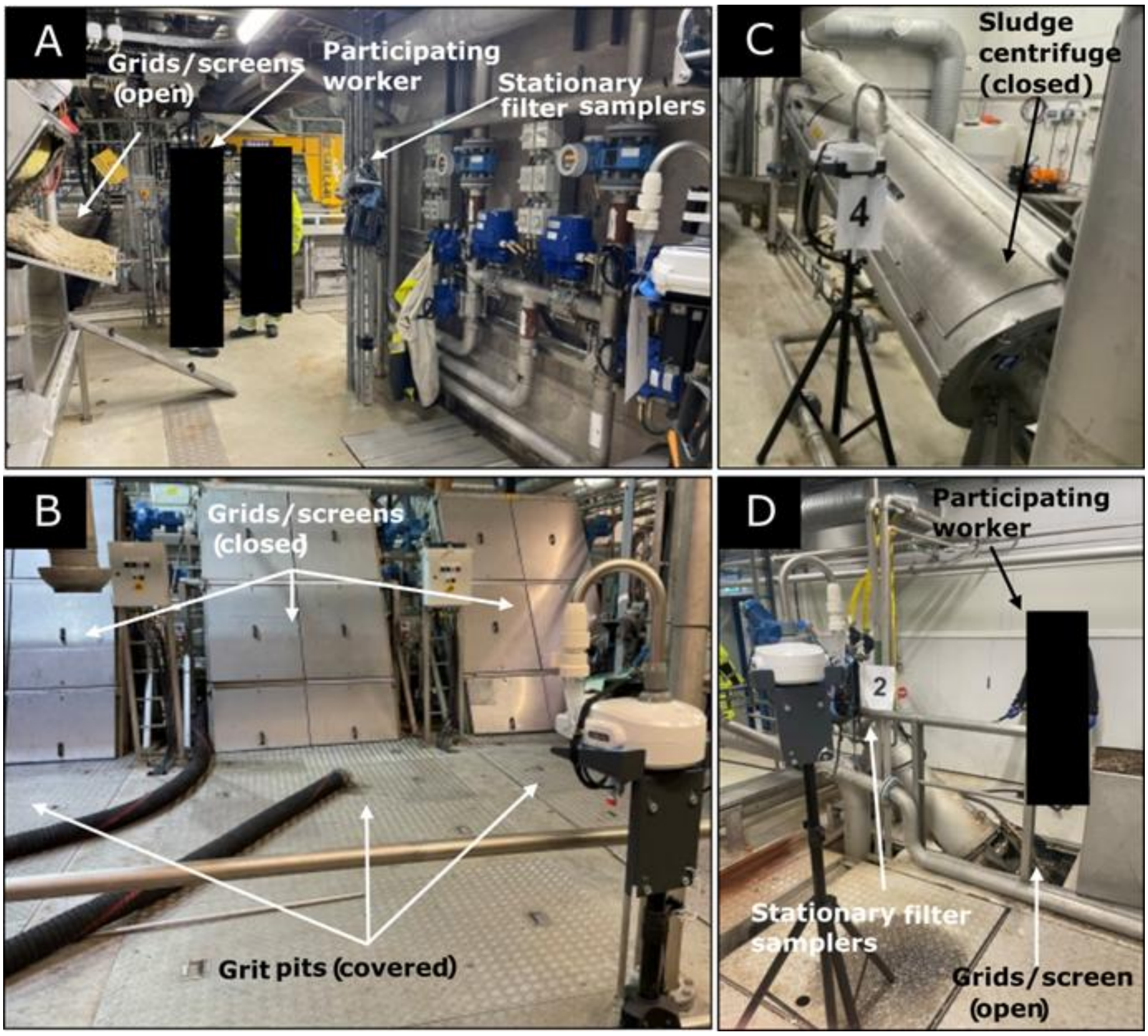

Fig. S1: Representative images of wastewater treatment processes in plants A, B and C. A) the grid station/grit chamber in plant A, B) the grid station in plant B, C) the sludge de-watering centrifuge in plant C, and D) the grid station in plant C

Table S1: The number of potentially exposed workers at each plant and the number of participants in our study. The treatment processes and tasks covered for stationary and personal air sampling campaigns are provided for each plant. For stationary samples, the treatment processes are numbered by their order in the treatment process. In Plant B, the sedimentation basin and sand filter are inside the processing hall and are presented with letters

	Plant A	Plant B	Plant C
**Potentially exposed / Participants**	22 / 7	43 / 12	1 / 1*
**Workstations covered by stationary air sampling**	1. Wastewater Intake<br>2. Grids<br>3. Flocculation Basins<br>4. Sedimentation<br>5. Sludge Treatment<br>6. Sand Filter<br>7. Biological Cleansing<br>8. Air Treatment	1. Grids/Grit pit<br>2. Flocculation Basins<br>3. Processing Hall<br>a. Sedimentation<br>b. Sand Filter<br>4. Sludge Treatment	1.Wastewater Intake/Grid<br>2. Sludge Treatment<br>3. Flocculation<br>4. Air Treatment Room
**Workstations covered by personal air sampling**	Grids<br>Sludge-treatment<br>Biological cleansing<br>Polymer room<br>Sludge building<br>Air treatment room<br>Basin halls	Wastewater tunnel<br>Grids/Grit pit<br>Sludge treatment<br>Biological cleansing<br>Basin halls<br>Processing hall<br>Sedimentation basin<br>Rottening tank	Water intake/Grid<br>Sludge treatment<br>Flocculation basin

*One worker in plant C was considered unexposed and included in the unexposed control group.

Table S2: PCR Primer sequences used in this study

Target	Primers and probes	Sequences	Ref.
**16S rRNA**	P11P F	5'GAGGAAGGTGGGGATGACGT 3'	[1]
	P13P R	5'AGGCCCGGGAACGTATTCAC 3'	
**IAV**	IAV F	5' GACCRATCCTGTCACCTCTGAC 3'	[2]
	IAV R	5' AGGGCATTYTGGACAAAKCGTCTA 3'	
	IAV P	5' TGCAGTCCTCGCTCACTGGGCACG 3'-MGBNFQ	
**Human AdV A-F**	JTXVF	5' GGACGCCTCGGAGTACCTGAG 3'	[3]
	JTXVR	5' ACIGTGGGGTTTCTGAACTTGTT 3'	
	JTXVP	6-FAM 5' CTGGTGCAGTTCGCCCGTGCCA 3'- MGBNFQ	
**bCoV**	BCoV 1F20	5'TGGTGTCTATATTCATTTCTGCTG 3'	[4]
	BCoV 1R89	5' GGCCACTGCCTAGGATACA 3'	
	BCoV 1P48	5'ACACGTCCCTGGCTGAAAGCTG 3'-MGBNFQ	

Table S3: Stock viruses used in this study and their origin.

Virus	Strain	Credit	Ref.
**AdV**	Human Adenovirus F40 strain Dugan	ATCC (ID VR-931)	[5]
**IAV**	Influenza A (H1N1) strain PR8	NMBU, Faculty of Veterinary Medicine	[6]
**bCoV**	Bovine Coronavirus	NMBU, Faculty of Veterinary Medicine	[7]
**MS2**	*Escherichia coli* bacteriophage MS2	NMBU, Faculty of Veterinary Medicine and ATCC (ID 15597-B1)	

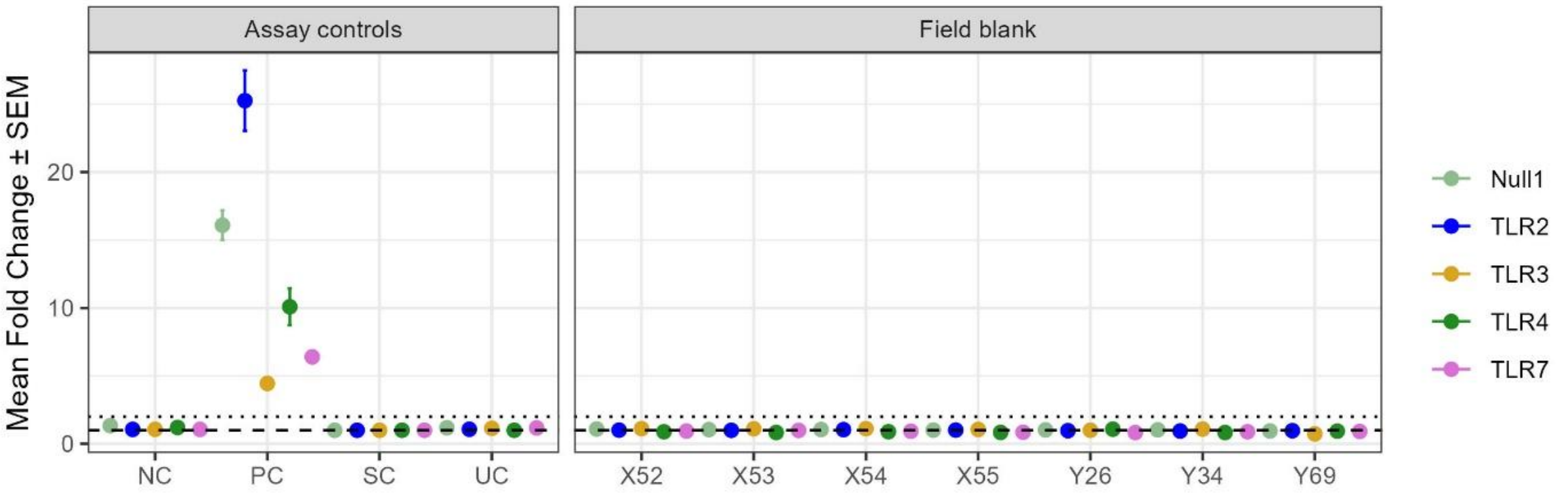

Fig. S2: Fold change TLR activation in positive (PC), solvent (SC), negative (NC) and untreated control (UC) and in field blanks in Null1 parental control cell line, HEK-Blue TLR2, 3, 4 and 7. The dashed line depicts fold change = 1 (no change) and the dotted line depicts fold change = 2 (doubling).

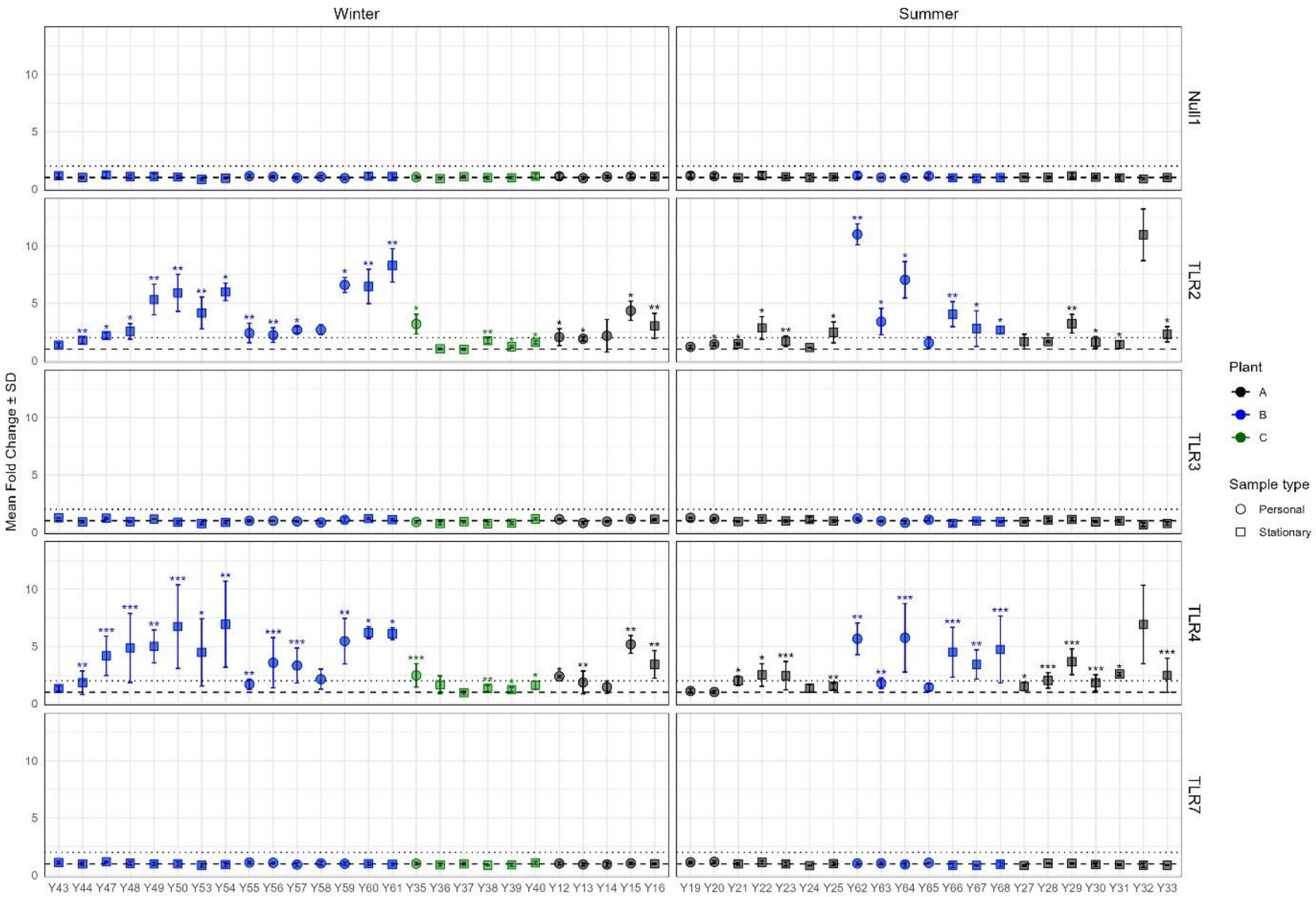

Fig. S3: Fold change activation of TLR2, 3, 4, 7 and the Null1 parental control line in all personal (circle) and stationary (square) samples collected. Each measurement is colored by the plant. Significant activation is indicated with asterisks (< 0.05, **p < 0.01, and ***p < 0.001). The dashed line depicts fold change = 1 (no change) and the dotted line depicts fold change = 2 (doubling).*

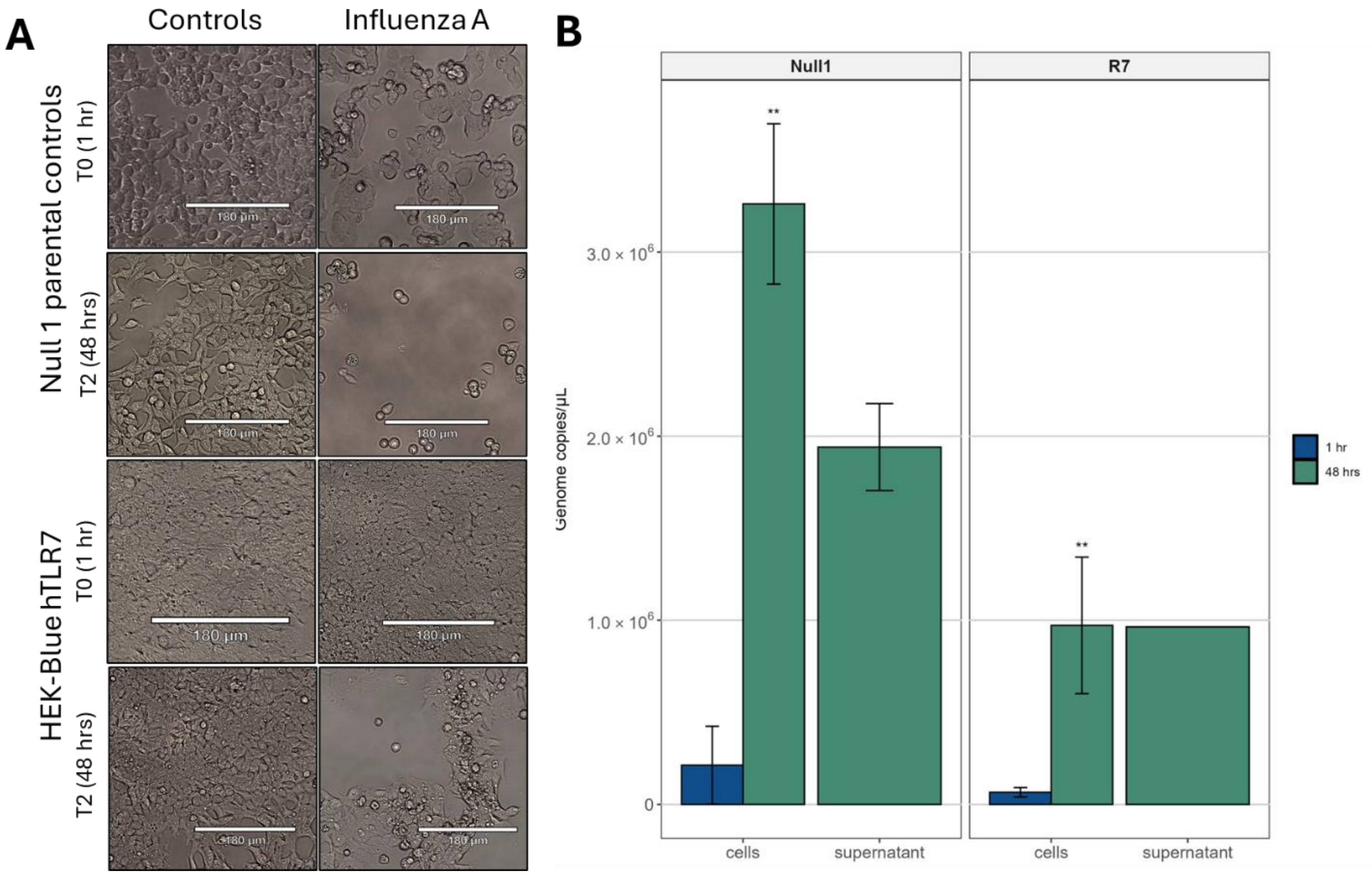

*Fig. S4: Morphological and genomic evaluation of the ability of IAV to infect HEK-Blue reporter cells. (A) Representative images of control cells (left panel) and cells inoculated with IAV one and 48 hrs after inoculation (right panel) in Null1 parental control cells (upper quadrant) and HEK-Blue hTLR7 cells (lower quadrant). (B) Genome concentration of IAV in cells and supernatant one and 48 hrs after inoculation. The data are presented as the average genome copies/µL sample (lysed cells or cell medium) and statistically significant differences between one and 48 hrs are marked with ** p<0.01.*

Table S4: Logistic model fit for health outcomes and adjusting variables. Each model was fitted with a generalized logistic model (glm, family = binomial) in R/Rstudio with exposure status dedicated by exposed or controls.

Outcome	Variable	OR (95% CI)	p-value	AIC	model
**Fever**	Intercept	0.05 (0 - 0.22)	0.000	46	$\beta_0 + \beta_1 * exposed + \varepsilon$
	Exposed (ref. Control)	15 (2.4 - 284)	0.010		
**Sneezing**	Intercept	0.3 (0.1 - 0.7)	0.010	62	$\beta_0 + \beta_1 * exposed + \varepsilon$
	Exposed (ref. Control)	5.4 (16 - 21)	0.010		
**Dry skin**	Intercept	0.35 (0.13 - 0.85)	0.030	64	$\beta_0 + \beta_1 * exposed + \varepsilon$
	Exposed (ref. Control)	4.3 (1.3 - 15)	0.020		
**Diarrhea**	Intercept	0.1 (0.01 - 0.36)	0.000	52	$\beta_0 + \beta_1 * exposed + \beta_2 * nicotine\ intake + \varepsilon$
	Exposed (ref. Control)	6 (1.3 - 43)	0.040		
	Daily nicotine intake (0/1)	1.8 (0.7 - 5.3)	0.270		
**Congested nose**	Intercept	0 (0 - 0)	0.005	45	$\beta_0 + \beta_1 * exposed + \beta_2 * BMI + \varepsilon$
	Exposed (ref. Control)	54.24 (6.1 - 1353)	0.003		
	BMI (continuous scale)	1.7 (1.2 - 2.8)	0.009		
**Sneezing**	Intercept	0.3 (0.1 - 0.7)	0.010	62	$\beta_0 + \beta_1 * exposed + \varepsilon$
	Exposed (ref. Control)	5.4 (1.6 - 21)	0.010		

Table S5: Logistic model fit for health outcomes by bacterial exposure measurements 16S rRNA gene copies (bact_DNA) and endotoxin levels (Endotoxin). Each model was fitted with a generalized logistic model (glm, family = binomial) in R/Rstudio.

Outcome	Exposure	Variable	OR (95% CI)	P-value	AIC	N	model
**Diarrhea**	Bacterial DNA	Intercept	30 (0 – 9.7x10^5)	0.47	33.7	22	$\beta_0 + \beta_1 * \log_{10} bacterial\ DNA + \varepsilon$
		Variable	0.5 (0.05 - 3.2)	0.45	33.7	22	
	Endotoxin	Intercept	0.3 (0.03 - 1.9)	0.25	26.08	17	$\beta_0 + \beta_1 * \log_{10} endotoxin/m^3 + \varepsilon$
		Variable	1.7 (0.59 - 5.9)	0.34	26.08	17	
**Fever**	Bacterial DNA	Intercept	3.5 (0 – 5x10^4)	0.78	34.21	22	$\beta_0 + \beta_1 * \log_{10} bacterial\ DNA + \varepsilon$
		Variable	0.7 (0.09 - 5.1)	0.75	34.21	22	
	Endotoxin	Intercept	1.2 (0.2 - 7.5)	0.84	26.51	17	$\beta_0 + \beta_1 * \log_{10} endotoxin/m^3 + \varepsilon$
		Variable	0.7 (0.22 - 1.9)	0.48	26.51	17	
**Headache**	Endotoxin	Intercept	1.6 (0.27 - 11.8)	0.59	23.94	17	$\beta_0 + \beta_1 * \log_{10} endotoxin/m^3 + \varepsilon$
		Variable	0.4 (0.11 - 1.3)	0.17	23.94	17	

Table S6: Spearman rank correlation coefficient (rho, ρ) between bacterial DNA (16S rRNA), endotoxin (EU/m^3) and fold change activation of TLR2 and TLR4 ($^{**}p < 0.01$, and $^{***}p < 0.001$).

	**Bacterial DNA**			
**Spearman rho (ρ)**	ρ = 0.48 **	**Endotoxin**		
	ρ = 0.29	ρ = 0.62 ***	**TLR2**	
	ρ = 0.09	ρ = 0.66 ***	ρ = 0.91 ***	**TLR4**

Questionnaire – exposed individuals

Exposure to viruses in different working environments

ID-code ☐☐-☐☐☐ Date (dd.mm.yyyy) . .

HEALTH PROBLEMS QUESTIONNAIRE

To answer the questions please choose the appropriate box. IF YOU ARE UNSURE OF YOUR ANSWER, **SELECT "NO".** For questions without a box option, try to describe the answer as best as possible. Spend plenty of time on your answer. It is very important for the results of the survey that you respond as precisely as you can.

Thank you for your help!

1. PERSONAL DATA

Male ☐ Female ☐ Other ☐

Age _______________ *years*

Height _________________ *cm* Weight _____________________ *kg*

2. QUESTIONS ABOUT THE USE OF SMOKE/SNUFF

2.1. Do you smoke cigarettes daily? ☐Yes ☐ No

To yes:

How many years have you smoked daily? Enter the number of years: __________

How many cigarettes do you smoke daily? Enter quantity per day: ______

2.2. Do you use snuff daily? ☐Yes ☐ No

To yes:

How much snuff per day? Enter the number of snuff packages /doses: _____

Strength: __________________

2.3. Do you currently smoke e-cigarettes daily? ☐Yes ☐ No

To yes:

What type of ampoules do you use?

☐ Without nicotine

☐ With Nicotine Strength:__________

☐ Without nicotine, with flavoring

☐ With nicotine and flavoring Strength:_________

Answered by those who do not smoke now:

2.4. Have you smoked daily before? ☐Yes ☐ No

To yes:

Has it been more than 1 year since you quit smoking? ☐Yes ☐ No

How many years did you smoke in total? Enter the number of years: _____

How many cigarettes did you smoke daily on average? Specify number per day: ______

3. CENTRAL NERVOUS SYSTEM AILMENTS

3.1. Do you have a headache at least once a week? ☐Yes ☐ No

3.1. Are you often dizzy? ☐Yes ☐ No

3.2. Have you fainted while working in the facility? ☐Yes ☐ No

Specify how often and what type of work task was done then.

____

4. ALLERGIES

4.1 Do you have allergies, e.g. against grass or animals? ☐Yes ☐ No

If yes, what you are allergic to: _________________________

If yes, what symptoms do you have/had?

a. Symptoms from the eyes ☐Yes ☐ No

b. Symptoms from the nose, sneezing and runny or stuffy nose ☐Yes ☐ No

c. Asthma ☐Yes ☐ No

d. Skin symptoms, eczema, or itchy rash ☐Yes ☐ No

Other: ____

5.	**ASTHMA**	
5.1.	Have you ever been diagnosed with asthma by a doctor?	☐Yes ☐ No
	If yes:	
	a. As a child or adolescent	☐Yes ☐ No
	b. As an adult	☐Yes ☐ No
	c. Still/Now	☐Yes ☐ No
5.2.	Have you had asthma attacks in the last 12 months?	☐Yes ☐ No
5.3.	Are you taking medication for asthma (inhalers, aerosols and tablets)?	☐Yes ☐ No

6.	**RESPIRATORY AILMENTS**	
6.1.	Which of the following statements best describes your breathing?	
	☐ I never / very rarely have problems with breathing	
	☐ I regularly have breathing problems	
	☐ My breath is never quite right	
6.2.	Have you had the flu, sore throat, eye inflammation and/or a cold in the last 12 months?	
	☐ Yes, 1-3 times ☐ Yes, 4-6 times ☐ Yes, more than 6 times	☐ No
6.3.	Have you had flu-like symptoms in the last 12 months, e.g. fever/chills that have passed within a couple of days?	☐Yes ☐ No
6.4.	Do you have at any point in the last 12 months had seizures of wheezing during the day without your effort?	☐Yes ☐ No
6.5.	Have you had wheezing or wheezing in your chest in the last 12 months?	☐Yes ☐ No
6.6.	Do you usually cough when you wake up in the morning?	☐Yes ☐ No
6.7.	Do you usually cough up mucus from your lungs when you wake up in the morning?	☐Yes ☐ No
6.8.	Do you have a daily cough with mucus 3 months or longer in a year?	☐Yes ☐ No

7. SKIN AILMENTS

7.1. Have you ever had hand eczema? ☐Yes ☐ No

7.2. Have you ever had eczema on your wrists or forearms? ☐Yes ☐ No

(except the inside of the elbows)

If you answered "no" to questions 7.1. and 7.2., go to question 7. 4.

7.3. Does your eczema improve during periods when you are away from your normal work (e.g. weekends, holidays or other longer absences)?

a. Hand eczema ☐Yes ☐No

b. Eczema on wrist/forearms: ☐Yes ☐No

7.4. Did you have eczema as a child? (Also known as atopic eczema) ☐Yes ☐ No ☐I don't know

Has a doctor made the diagnosis? ☐Yes ☐ No

When was the diagnosis made? _______________ (age)

8. OTHER DISEASES AND MEDICATIONS

8.1. Have you had diarrhea/loose stools in the last 12 months with suspected infection in the workplace?

☐Yes, 1-3 times ☐ Yes, 4-6 times ☐ Yes, more than 6 times ☐ No

8.2. Have you or have had a serious or chronic illness (e.g., COPD, heart attack, coeliac disease, irritable bowel, Ulcerous Colitis, or Crohn's disease)? ☐Yes ☐ No

Specify which and whether you are still ill: ______________________________

8.3. Do you use prescription medications? ☐ Yes ☐ No

Specify which: ____

9. QUESTIONS ABOUT SYMPTOMS AND AILMENTS ASSOCIATED WITH THE WORK THE LATEST WEEK (Both while at work and within a couple of hours of

has ended for the day).

	Yes, often	Yes, sometimes	No
Eczema on the hands	☐	☐	☐
Dry skin on your hands	☐	☐	☐
Runny, itchy, sore, red eyes or eyework	☐	☐	☐
Fever	☐	☐	☐
Sneezing	☐	☐	☐
Stuffy nose	☐	☐	☐
Runny nose	☐	☐	☐
Sår hals	☐	☐	☐
Cough with mucus	☐	☐	☐
Cough with spit	☐	☐	☐
Wheezing in the chest	☐	☐	☐
Tightness of the chest	☐	☐	☐
Vomiting or nausea	☐	☐	☐
Headache	☐	☐	☐
Abnormal fatigue	☐	☐	☐
Loose stools	☐	☐	☐
Diarrhea	☐	☐	☐

References

1. Widjojoatmodjo MN, Fluit AC, Verhoef J Molecular identification of bacteria by fluorescence-based PCR-single-strand conformation polymorphism analysis of the 16S rRNA gene. J Clin Microbiol 1995; 33: 2601-2606.

2. Brisebois E, Veillette M, Dion-Dupont V, Lavoie J, Corbeil J, Culley A *et al* Human viral pathogens are pervasive in wastewater treatment center aerosols. J Environ Sci 2018; 67: 45-53.

3. Jothikumar N, Cromeans TL, Hill VR, Lu X, Sobsey MD, Erdman DD Quantitative real-time PCR assays for detection of human adenoviruses and identification of serotypes 40 and 41. Appl Environ Microbiol 2005; 71: 3131-3136.

4. Christensen E, Myrmel M Coagulant residues' influence on virus enumeration as shown in a study on virus removal using aluminium, zirconium and chitosan. J Water Health 2018; 16: 600-613.

5. de Jong JC, Wigand R, Kidd AH, Wadell G, Kapsenberg JG, Muzerie CJ *et al* Candidate adenoviruses 40 and 41: fastidious adenoviruses from human infant stool. J Med Virol 1983; 11: 215-231.

6. Taylor AR, Sharp DG, Beard D, Beard JW, Dingle JH, Feller AE Isolation and Characterization of Influenza A Virus (PR8 Strain)1. J Immunol 1943; 47: 261-282.

7. Oma VS, Traven M, Alenius S, Myrmel M, Stokstad M Bovine coronavirus in naturally and experimentally exposed calves; viral shedding and the potential for transmission. Virol J 2016; 13: 100.
